# Can Children of Different Ages Recognize Dog Communication Signals in Different Situations?

**DOI:** 10.3390/ijerph17020506

**Published:** 2020-01-13

**Authors:** Petra Eretová, Helena Chaloupková, Marcela Hefferová, Eva Jozífková

**Affiliations:** 1 Department of Ethology and Companion Animal Science, Czech University of Life Sciences Prague, Kamýcká 129, 165 00 Prague, Czech Republic; eretova@af.czu.cz (P.E.); vratislavicem@seznam.cz (M.H.); 2 Department of Biology, J. E. Purkyne University in Usti nad Labem, Za Válcovnou 1000/8, 400 96 Usti nad Labem, Czech Republic

**Keywords:** dog, children, emotion recognition, contextual cues, inner state assessment, audio communication, audio-visual communication

## Abstract

The presented study examines the ability of 265 children aged 4–12 years to correctly assign contextual cues and inner state values to a set of audio and audio-visual recordings of dog vocalizations and behaviors in different situations. Participants were asked to mark which situation each recording captured, what inner state of the dog it showed, and what inner state a human would feel in the same situation. Recognition of the inner state of dogs was affected by the age of the child when evaluating the audio recordings (*p* < 0.001), and such a tendency was revealed in evaluating the audiovisual materials (*p* = 0.08). The inner state of dog evaluation was associated with both the situation assessment (*p* < 0.01) and human inner state (*p* < 0.001) in the case of audio recordings, but it was only correlated with situation assessment in audio-visual recordings (*p* < 0.01). The contextual situations were recognized by the participants only in the audio materials, with “stranger” being the best recognized situation, while “play” was the least recognized. Overall, children aged 4–5 years showed a limited ability to understand dog signals compared to children aged 6–12 years, who were successful in recognizing the dogs’ stimuli more than 80% of the time. Therefore, children younger than 6 years of age require increased supervision when interacting with dogs.

## 1. Introduction

A long-term study in the United States [1] has shown that children under 10 years of age are the most common victims of dog attacks, and they are most likely to sustain fatal injuries. Beck and Jones [2] reported that children were most often bitten by a dog familiar to them, either belonging to their own family or to a neighbor. The same phenomenon was reported by Guy et al. [3]. One of the cited reasons for this is the potentially under-developed ability of preschool children to correctly recognize communication signals from dogs. An example of this is the significant difference in meaning of exposed teeth in people and dogs, i.e., what might look like a smile to a young child can actually be a serious threat. A study on children aged 3, 4, and 5, using audio and audio-visual materials and showing specific stress behaviors of dogs without context, revealed that uneducated children in this age range lack an understanding of canine stress signals and interpret a majority of them as signs of playfulness or affection [4]. Regarding audio cues, one study [5] confirmed the ability of children aged 5–10 to differentiate nonhuman vocal signals of the stump-tailed macaque (*Macaca arctoides*) and to identify basic inner states such as aggression, fear, a general “positive feeling”, as well as submission and dominance; this suggests an ability to understand universal mammal acoustic cues.

Playback experiments [6,7,8] revealed that adult humans are capable of categorizing dog vocalizations without having previous experience of owning a dog. Even children as young as 6 years of age can correctly point out dogs barking at a stranger and recognize such barks as being more aggressive than others [8], which reflects the importance of being able to recognize aggression during early childhood. Barks recorded while the dogs were left alone were generally considered more fearful by the respondents. Overall, there was no significant difference between the age groups (5, 8, and 10 years) when it came to matching the inner states of dogs to each recording [8]. Nevertheless, the level of such abilities in naive preschool children remains largely unclear, and seems to have been neglected so far as an area of study. In addition, previous studies have mainly focused on the effects of acoustic signals [8] on individual behavioral patterns without introducing context in order to intervene and educate [4,9]. Information regarding the combination of visual and acoustic signals in the context of a situation, or the comparison of the various nature of the signals, is largely missing.

The objectives of the study were to utilize audio and audio-visual recordings to determine the following: (i) the effect age has on the child’s ability to recognize both dog stimuli and the typical situations in which the dogs were recorded; (ii) whether children could match inner states of the dogs to those of typical human emotions, as signaled through facial expressions; and (iii) if their performance is affected by their dog ownership status. We predicted that younger children would possess a decreased ability to correctly recognize all stimuli regardless of their level of experience with dogs at home.

## 2. Materials and Methods

### 2.1. Ethical Approval

The study was performed in accordance with relevant guidelines and regulations. Data collection was preceded by the provision and signing of informed consent forms. The parents of the participating children were given a detailed explanation of the applied methods through each institution’s headmaster. The data were collected anonymously. The recordings of the dogs were made during routine life situations, with the ethical committee of the Czech University of Life Sciences specifically stating that this did not constitute an animal experiment according to legislation of the Czech Republic. The protocol was approved by the Czech Central Committee for the Protection of Animals (Permit No.: 63479/2016-MZE-17214).

### 2.2. Data Collection and Participation

Data collection took place in 2017 in the north of the Czech Republic, near the cities of Liberec and Jablonec nad Nisou. For the data collection on the basis of the audio recordings alone, a total of 265 children participated. These children were aged 4–12 years and attended either one of three kindergartens or two elementary schools. Of these children, 152 also participated in the data collection on the basis of the audio-visual recordings (one elementary school and one kindergarten declined to participate in the audio-visual experiment for administrative reasons). The children were visited in their classrooms by the experiment coordinator and divided into groups of five in the kindergartens or into groups of eight in the elementary school. The small kindergarten group was necessary to facilitate understanding of the questionnaire. However, the teachers were careful not to provide input so as not to affect the children’s performance in any way.

### 2.3. Questionnaire and Procedure

**Figure 1:**
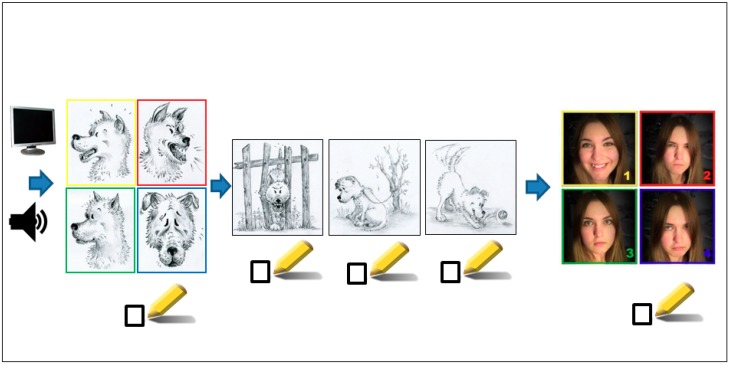
Experiment design. The recordings (either audio or audio-visual) were played back to the children. They were subsequently asked to fill in the questionnaire by using color pencils to mark: the dog’s inner state by choosing one of four drawings reflecting the mood of the dog; the situation captured on the recording by choosing one of three drawings; and the expected inner state of a human in the same situation by choosing one of four photographs of a young woman with differing facial expressions.

The first step involved handing out the questionnaire to the children and giving instructions on how to fill in the questions regarding their age, gender, and dog ownership status. This was followed by a detailed explanation of the inner states captured in drawings of a dog and photographs of a young woman, and the task itself. These two sets of images were color-coded to provide ease of understanding to the participants (red for “angry”, yellow for “happy”, blue for “sad”, and green for “fearful”), and children used crayons of the corresponding color to mark each inner state. The preschool children identified only one audio and one audio-visual recording. The audio-visual recording always matched the same audio recording for the same question. However, the children were not told that both types of recordings were taken in the same situation and at the same time. The recordings were then played back to the children. They were subsequently asked to mark what situation was taking place by identifying a single illustration out of a set of illustrations of dogs in three situations: alone, playing, barking at a fence. Participants were allowed to mark their answers using crayons, but the colors were not taken into account. Furthermore, using age-appropriate phrases such as “what is this dog feeling right now?” or “if this girl was the dog, what would she feel?”, the children were asked to determine the dog’s inner state (sadness, fear, joy, anger) as presented in a set of four drawings of dogs and a set of four photographs of a young woman with different facial expressions (see Figure 1).

The “younger” children were played only one recording and asked to choose which situation and inner states they felt were the most appropriate. This was done due to their short attention span and to acclimatize their cognitive abilities. The children were generously praised for each answer, regardless of whether it was correct or incorrect. At no point during the experiment were the children told the correctness of their answers. The maximum time allotted for the procedure was 15 min.

The validity of both sets of drawings regarding “inner state” (dog’s inner state, situation) and the set of photographs (human facial expression) were tested on multiple sets of adults and children alike to confirm the clarity of their meaning.

### 2.4. Audio and Audio-Visual Playback

Five dogs of 4–9 years of age (average 6.1 years) were recorded in three typical situations. Two dogs were male (a German shepherd and a Pit-bull/Dachshund mix), three were female (a German shepherd, Staffordshire terrier/Belgian Malinois mix, and a Cardigan Welsh Corgi). The first recorded situation was “play”, in which the dogs were recorded while playing with each other or the experiment coordinator. The second situation was “alone”, in which the dogs were left in the presence of the experiment coordinator while their owner walked away. The last situation was “stranger”, in which the dogs were recorded while barking at an unknown person at a fence or gate (see Table 1).

For both the audio and audio-visual recordings, the dogs were recorded in their domestic environment or close surroundings, with the owner present if the situation so permitted. All the dogs included in the study were kept as house pets and the owners participated in the research voluntarily. No dogs were filmed twice. However, the vocalization of the Cardigan Welsh Corgi female was recorded during “play” and “alone” situations and later used in the study.

### 2.5. Editing of Collected Materials

The recording device used to gather both the audio and audio-visual recordings was an iPhone SE (Apple, Inc., made in 2016). Only audio recordings that bore no external noise corruption were included in the study. All collected audio recordings were digitized. Each audio recording consisted of several vocalization sequences from the aforementioned dogs. All the segments were digitally cleaned of distractive background noises and then put together into one single composite track for each of the three situations. The audio recordings were manipulated by cutting out long silent phases and sequences of people talking in order to shorten their duration, and subsequently lengthened to last 30 s by including multiple individual bark sections. The purpose of this exercise was to make the recordings more straightforward and understandable. The acoustic structure and temporal patterning of the recordings were not manipulated; thus, the reflection of their natural variation remained intact. Other than cutting sequences in which the dogs were being idle, the audio-visual recordings were not manipulated in any way. The final audio-visual recordings were 5 s long.

In order to avoid unintentional bias, the person in charge of creating the recordings was not the experiment coordinator, i.e., the person who played back the recordings to the participants. In fact, they never met any of the recorded dogs, and were therefore able to maintain neutrality within the context of the recordings.

### 2.6. Statistical Analysis

The data were analyzed with SAS (SAS Institute, Inc., Cary, North Carolina, USA; version 9.4) using Generalized Linear Mixed Models (GLMM, proc GLIMMIX). The results were considered statistically significant if *p* ≤ 0.05.

**Figure 2:**
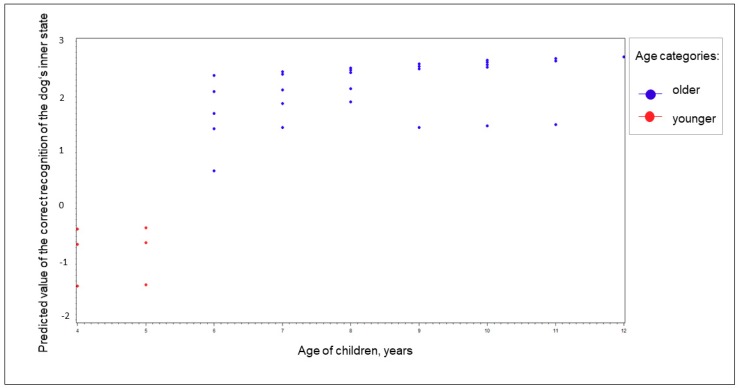
Preanalysis of the effect of age on the correct recognition of the dog’s inner state divided by age category in audio recordings.

During the preanalysis, we tested the effect of age (F_1, 32,33_ = 0.01; *p* = NS) and age category (F_1, 95,65_ = 8.07; *p* = 00.006), i.e., “younger” (4 and 5 year olds) and “older” (6–12 year olds), on the ability to recognize the dogs’ inner state from the audio recordings (Figure 2). This categorization supports previous studies into the rapid development of a child’s ability to recognize inner states around the age of 6 and the need to explore the existing ability to differentiate inner states in children on both sides of this age mark [8,10,11,12].

Finally, we tested how the age category of the children (“younger” vs. “older”) affected the ability to correctly assign the dogs’ inner states from the audio playbacks and/or from the audio-visual recordings in relation to the ability to assign the “human’s inner state” and “situation”. The “dog ownership” (yes/no) was used as a fixed effect in all models. The fitted models included an interaction between class and school identity as a random effect to account for the use of repeated measures on the same individuals. The models were estimated using a fit statistic (Akaike’s Information Criterion, AIC) to increase the power of the statistical tests. A model with a minimum value of AIC is chosen to be the best fitting model among several competing models. Therefore, variables “child gender”, were excluded from the model because of the nonsignificant effect in all models.

Least square means were calculated by computing the mean of each treatment (class) and averaging the treatment (class) means. These means of means were then used to compare the factors. In this way, the means were adjusted for the number of observations in each treatment. This estimate is unbiased because of the unequal number of observations taken into account. Least square means (also referred to as ‘‘adjusted means’’) were computed for each class, and the differences between the classes were tested using the *t*-test. Tukey-Kramer adjustment was used for multiple comparisons.

## 3. Results

The number of participating children by gender and dog ownership is shown in Table 2.

### 3.1. Audio Recordings

**Figure 3:**
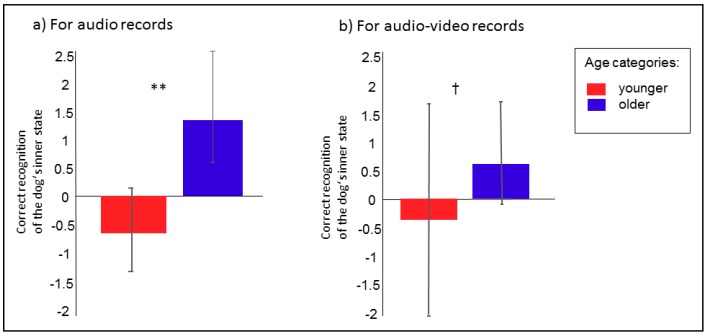
(**a**,**b**) Probability of correctly assigning the dog’s inner state from audio (**a**) and audio-visual (**b**) recordings according to age category. LS means, 95% CI, ^†^ *p* < 0.1; ** *p* < 0.01.

**Figure 4:**
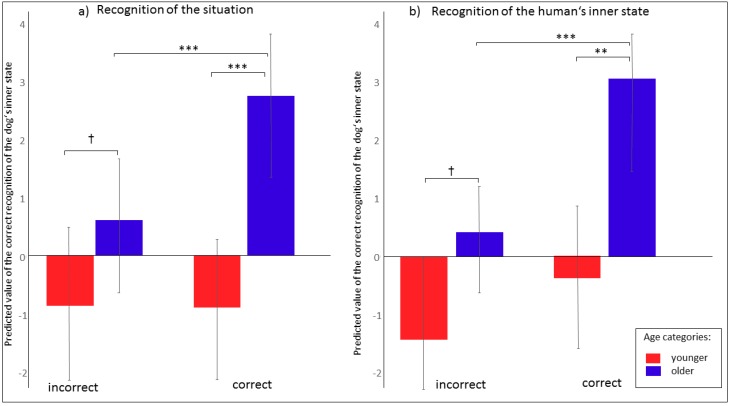
(**a**,**b**) The ability to assign (**a**) the situation and (**b**) the human’s inner state in relation with the correct recognition of the dog’s inner state from audio recordings according to age category. LS means, 95% CI, ^†^ *p* < 0.1; ** *p* < 0.01; *** *p* < 0.001.

**Figure 5:**
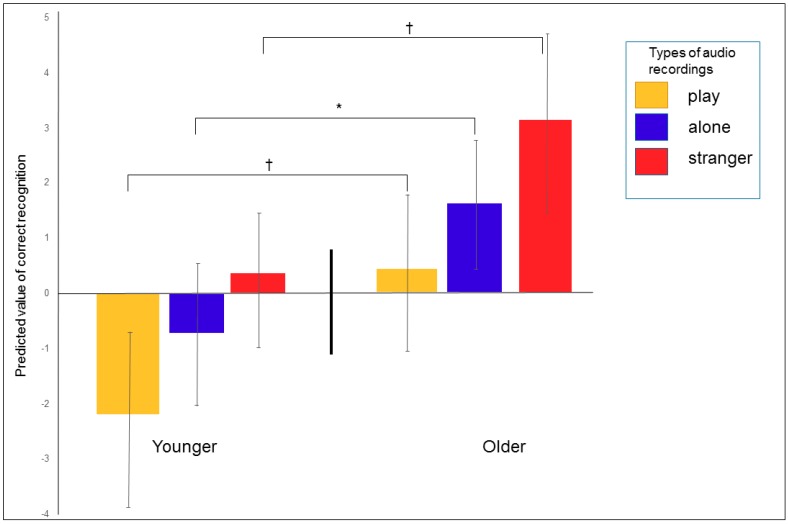
Success rate in determining the situational context from audio recordings according to age category. LS means, 95% CI, ^†^ *p* < 0.1; * *p* < 0.05.

The age of the children significantly affected their ability to correctly assign the dogs’ inner states (F_1, 57.49_ = 15.75; *p* = 0.0002). Among the younger children, the probability was lower that they would correctly assign audio playbacks compared to the older children (Figure 3a). A strong positive relationship was also found with regards to the probability of correctly assigning the same playback to the recorded situation of the dog barking (Figure 4a; F_2, 320_ = 5.96; *p* = 0.003) and to the human’s inner state (Figure 4b; F_2, 320_ = 10.41; *p* = 0.0001). In all cases, the older children were more successful. The children significantly differed in their ability to correctly recognize the type of audio recording (F_4, 320_ = 3.89; *p* = 0.004). In general, the “stranger” audio recording was the most successful, followed by “alone” and “play” (Figure 5). Dog ownership was found to have no significant effect (F_1, 320_ = 0.93; *p* = 0.33).

### 3.2. Audio-Visual Recordings

**Figure 6:**
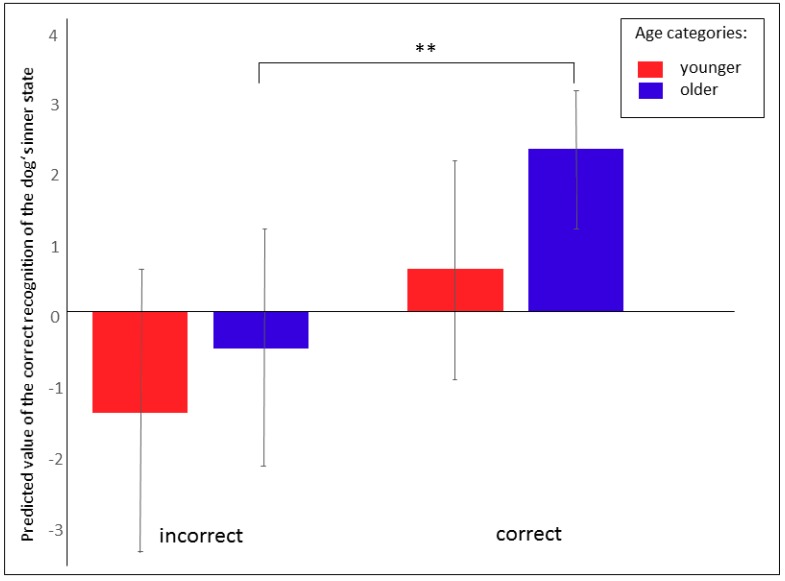
The ability to assign the situation in relation with the correct recognition of the dog’s inner state. LS means ± 95% CI, ** *p* < 0.01.

Only a tendency towards differences between the younger and older children in their ability to correctly assign the dog’s inner state (F_1, 40_ = 3.13; *p* = 0.08) was found, whereby the older children tended to be more successful than the younger ones (Figure 3b). As was the case for the audio recordings, a positive relationship was detected between age category and the probability with which they were able to assign the dog’s inner state and the recorded situation (Figure 6; F_2, 147_ = 6.69; *p* = 0.002). Dog ownership was found to have no significant effect (F_1, 147_ = 0.58; *p* = 0.45). No significant relationship between the ability to assign the dog’s inner state and the human’s inner state was detected (F_1, 1_ = 6.93, NS).

## 4. Discussion

The ability of children to differentiate dogs’ inner states using only audio recordings of dog vocalizations was proven. Based on the varying levels of success among the age categories, it is possible to estimate how age affects children’s perceptions of dog communication signals, as well as to measure the effectiveness of given signals and identify those that are the easiest to understand.

The children aged 4–5 years fared almost 60% worse than children aged 6–12 years when evaluating the audio recordings. Similarly, they tended to fare worse by 45% when evaluating the audio-visual recordings. The success rate of children who owned a dog did not differ from their non-dog-owning peers. In general, the children were capable of: (a) matching pictured inner states to dogs based on playback alone; (b) determining which situation was taking place; (c) matching the dog’s inner state with the corresponding inner state of a human face from a set of photographs. They therefore showed the ability to understand the dog’s inner state. It appears that in the given situations, the dogs emitted signals that were understandable to the participating children, and that the level of canine inner state understanding depends on the age of the child, and therefore, on the stage of ontological development.

In the case of the older children, a previous study [8] found a similar high ability to understand audio signals. In the case of the younger children, only a few studies [4,13,14,15] have tested the positive effect of teaching on the success rate of recognizing canine communication signals. On the other hand, no knowledge about their naive ability has been presented. A study by Meints et al. [4] revealed that conflict-escalating signals (i.e., growling, snapping, and biting) in particular were the clearest to children aged 3–5 years, and are the most easily taught to interpret correctly. The results of our study confirm this, whereby audio signals perceived as aggressive were the easiest for the participants to decipher. This reflects the need for children to recognize dangerous situations and know when to avoid interactions. Meints et al. [4] also reported a high success rate in teaching children aged 3–5 years and their parents about the true meaning of such signals and the retention of that information for over a year after the initial explanation. This shows that an early intervention and the education of young children and their parents may eliminate a significant number of disastrous confrontations between children and domestic dogs.

The children were also found to be able to correctly assign contextual situations to the audio recordings, with their performance improving with age. This is in accordance with the findings of Pongrácz et al. [8], and shows that with age, children get better at identifying situations from dog vocalizations. A positive relationship was also found between the ability to identify a dog’s inner state, situation, and a human’s inner state from the audio recordings. This supports the theory that once a child correctly identifies the dominant inner state from a dog’s bark, it can better analyze the situation and deduce the context. The experiment showed that the children could successfully relate the dog’s inner state to that of a human, thereby demonstrating their ability to connect and show empathy with dogs.

The most successfully rated audio recording was the one that captured the dogs’ responses to a “stranger”, followed by “alone” and “play”. It may be somewhat surprising that the recording generally rated as the most fearful was the second best recognized, because the developmental pattern for fear is one of the slowest to emerge in childhood [15]. However, it is important to mention that in humans, the perception of positive and negative inner states varies according to the form of input, i.e., positive inner states tend to be more understandable when presented visually, while negative inner states are more relatable acoustically [16]. This could mean that the audio recording capturing “play” was, due to its lack of visual clues, the most challenging for children to rate. Further detailed research may help to clarify this issue.

A significant difference in perception between the audio and audio-visual recordings was not supported. The participants in the study achieved similar success rates for all three tasks for both types of recordings. The answer to the question of why recording with a wider variety of inputs (video) did not provide a fundamental advantage compared to recordings of a limited character and input (audio) is up for debate. It is possible that the children in our study were not yet old and mature enough to properly understand the visual cues from the dogs because the ability to properly interpret conspecific visual signals develops from early infancy and typically does not hit a meaningful success rate until around two years of age [17], with allospecific signals possibly taking even longer to fully form. Another possible solution to this enigma may be that the message collected from the audio part of the videos proved to be descriptive enough to render the visual side of the audio-visual recordings redundant. As dogs use the same signals to interact with humans as with other dogs, i.e., following the structure-acoustic rules defined by Morton [18], it is conceivable that the structure of the recorded vocalizations itself revealed more information about the dogs’ inner states and the situations they were in at the time the recordings were being made that the children omitted the visual stimuli for the raw sound. Russell and Widen [19,20] suggest that children of 2–7 years of age prefer auditory input over visual input when assigning inner states to situations or other living beings, which is in accordance with our findings. Tami and Gallagher [21] reported that when deprived of audio input from videos of dog behavior, the participants (including children) in their study had to rely on the facial expressions of said dogs, which proved to be less reliable and made it more difficult to identify aggressive behavior; interestingly, fearful behavior, as opposed to other behaviors, was the most easily recognized in this study [8,9]. The key, it seems, is the absence of sound. As mentioned above, the character of the recordings and the captured inner state of the dog could be the key to rating success. As some dogs favor visual signs of preaggressive behavior (i.e., baring teeth, pulling back ears, raising their hackles) to vocalization (i.e., barking or growling), this puts many children at a greater risk of misunderstanding dogs’ behavior, and therefore, the risk of attack. As the study presented here only made use of materials equipped with sound, it proves that aggressive dog vocalizations are quite understandable, even to children around 4 years of age.

The older children were more likely to correctly assign the expected inner state to both the audio and audio-visual recordings—a pattern which has also been confirmed by other authors [4,8,9,14]. Lakestani et al. [4] reported that the 430 children included in their video playback study showing dog behaviors showed an increasing ability to correctly interpret dog communication signals with age, whereby fearful behavior was the most puzzling and aggressive behavior the clearest to all participants. Our results support this phenomenon. From an evolutionary point of view, it makes absolute sense that children would first learn when a dog is most likely to turn aggressive, and therefore, endanger their health and life.

Concerning the video rating experiment, the children in the “older” category showed, compared to the “younger” children, a tendency to achieve an increased success rate to correctly assign the dog’s inner state and the situation. As stated above, it is natural for humans to differentiate inner states based on the character of the input [16], and that when faced with purely visual images of aggressive dogs, people tend to be confused and unsure of the true nature of the situation [21]. As our recordings combined sound with images, it is possible to theorize that some participants struggled with some aspects of the videos (such as dogs showing their teeth and mock-fighting in the “play” situation, or a dog wagging its tail rapidly in the “stranger” situation), which led to inconsistencies in their assignment.

Interestingly, the children in this study also showed a tendency to correctly assign the human’s inner state to each video. However, what is puzzling is that no association between the assessment of the dog’s inner state and that of the human’s inner state was found. This may be explained by the lack of a proper connection between the children and the dogs. As already stated, and in accordance with previous findings [4,8], especially the younger children had trouble assessing the situation and the dog’s inner state, such as mistaking an aggressive animal for a happy and playful one. It is possible that the task might have been too abstract for the children, and that they failed to fully connect with the dogs and put themselves into their situation. Another possible explanation might be that the children did connect with the dogs, but took the task too literally and marked what inner states humans would be showing using the same facial expressions as the dogs in the audio-visual recordings. This would mean that they perceived the smile-like bared teeth in the “stranger” situation or the playful scowl in the “play” situation as happiness and anger, respectively, thus exhibiting a form of inner state contagion using mirror neurons [22]. In order to properly clarify the issues surrounding the understanding of the audio-visual signals of dogs in young children, a more robust study using a larger selection of audio-visual recordings with a greater variety of dog breeds is necessary.

No significant difference was found between the performance of dog-owning children and that of their peers with no domestic experience with dogs; this finding corresponds with previous conclusions made by Meints et al. [4] and Pongrácz et al. [8]. Since this study could not provide an equal number of dog-owning and non-dog-owning children for each age category, dog ownership could have played a larger role which remained undisclosed. Lakestani et al. [9] suggest that a difference in performance between dog owners and people without dogs surfaces in adulthood, with dog owners being more adept at assessing dog signals than others. This could mean that adults have typically spent a significant enough measure of time around dogs to gain advantage from this experience, while children have yet to reach this level of learning. Additionally, it was found that older participants provided a more robust performance and scored a higher number of correct answers than their younger counterparts. Both findings confirm the results of previously published research [4,8,9].

## 5. Limitations

The study’s design allows for some limitations, such as the possible occurrence of an order effect bias, as the participants who rated both audio and audio-visual materials were tasked with assessing recordings portraying the same situations. This risk of order effect bias was minimized because the participants were not aware that the situations were the same. Another possible limitation would be the use of two dogs (a Welsh Corgi and a Pitbull-Dachshund mix) in the “play” audio-visual recording, whereas two German shepherds were used individually for the other two audio-visual recordings. The fact that two small dogs were used in one recording while two large dogs were used separately in the remaining two might have had some influence upon the audience. The study’s design never offered the participants a chance to become familiar with the other materials, thus preventing bias or the use of elimination in rating the materials.

Another point of limitation worth mentioning was the imbalance in the length of the presented material—the audio recordings were 30 s long, while the audio-visual recordings were only 5 s long (due to technical issues). However, the clarity of all materials was checked on multiple sets of adults and children to ensure that despite the short length of the videos, the message was clear. Additionally, during data collection, recordings were replayed for ease of understanding, greatly decreasing the effects of their insufficient length.

## 6. Conclusions

This study supports the existence of children’s ability to correctly classify dog behavioral cues, which develops after the age of 6. Our study showed that children younger than 6 exhibited a much more consistently flawed performance, which suggests a limited ability to interpret dog behavior and recognize contextual cues. In the case of assessing audio tracks, it was revealed that once the participants managed to correctly identify the dog’s inner state, they were more likely to correctly identify the situation and human inner state. When tasked with assessing the audio-visual recordings, the children could make a connection between the situational context and the inner state of the dogs, but could not associate the dog inner state with human inner states, indicating a potentially limited understanding of combined audio-visual signals. On a side note, children under 6 years of age should be closely monitored when interacting with dogs in order to ensure their safety.

## Figures and Tables

**Table 1:** Description of the dogs’ interactions during the recordings.

Situation	Inner State	Audio	Audio-Visual
Play	Happy	Neutered male Pit-bull/Dachshund mix; Intact female Cardigan Welsh Corgi	Neutered male Pit-bull/Dachshund mix and intact female Cardigan Welsh Corgi play-fighting together (filmed from upper-side view);
Alone	Sad	Intact female Cardigan Welsh Corgi; Intact female black-and-tan German shepherd	Intact female black-and-tan German shepherd barking and whining at departure of owner while in the presence of the coordinator (filmed from side view);
Stranger	Angry	Intact male black German shepherd; Intact female Staffordshire terrier/Belgian Malinois mix	Intact male black German shepherd barking at the coordinator from behind a fence (filmed from front view).

**Table 2:** Number of participating children divided by age category, gender, and dog ownership.

Recordings	Age Category	No. of Children Per Category	No. of Dog-Owners Per Age Category	Age	No. of Boys	No. of Girls
**Audio**	younger	55	20	4	8	10
				5	16	21
	older	127	30	6	14	10
				7	10	7
				8–12	45	41
**Audio-visual**	younger	26	10	4	3	4
				5	8	11
	older	127	30	6	14	10
				7	10	7
				8–12	45	41
